# Seventh Annnal Report of the Registrar-General of Births, Deaths, and Marriages, in England. (Abstracts of the Two Years, 1843, 1844)

**Published:** 1847-01-01

**Authors:** 


### Seventh Annual Report of the Registrar-General of Births, Deaths, and Marriages, in England. (Abstracts of the Two Years, 1843, 1844). 8vo. pp. 350. London, 1846.

It is surprising that a so practically sagacious people as the English should so often do things by halves. We have now for some years had an admirable system of Registration in operation, the results of which contained in the Annual Reports are quoted almost daily by British and foreign writers; and yet the advantages derivable from it have not been extended to Ireland and Scotland, containing a population of between ten and eleven million. If from the present data valuable conclusions are obtainable, how much more would this be the case when these became nearly doubled? We never could understand why what has been done for England and Wales was not attempted in the first instance for the United Empire. It required no very expensive organization, and shocked no prejudice; and even if greater difficulties were to be apprehended in endeavouring to work the measure in Ireland than have been found to prevail here, they should, on account of the great benefits which must accrue from vanquishing them, have been met, not avoided. We are pleased to find the Registrar-General, in his present Report, drawing the attention of the authorities to this important point: and hope that another session will not be allowed to pass by without the blunder being repaired. The able analysis of the Irish Census Returns, by Mr. Wilde of Dublin, and Dr. Stork's spirited exertions at Edinburgh, in framing the Bills of Mortality of that city, show that zealous and efficient coadjutors will not be wanting in either of those capitals. The Registrar-General thus expresses himself, addressing the Home Secretary.

"I submit to you that the Marriages, Births, and Deaths of the people of Scotland, Ireland, and England should all be registered on a uniform plan; and that the inquiry, which has already been so successful and beneficial in England, into the causes of death, should be extended to Ireland and Scotland. Like the institution of the Coroner's Jury, this inquiry deters from crime, fosters a reverence for human life, and by discerning the causes of premature death in the various circumstances of the population, will contribute to the progress of the science of medicine, diminish suffering, and lead to the prolongation of human life to its natural term. No argument that I am aware of can be used in favour of Registration which does not apply to Scotland and Ireland." P. 23.

The present Report will not require a long notice at our hands, inasmuch as it contains little else than the tabulated details, and a much smaller number of these than usual. One admirable feature in the working of the Registration Act is the fact that its administrators are never content with the *statu quo*, but are ever desirous of augmenting the amount or increasing the value of the information obtainable through its agency. Thus, we find that it is in future intended to furnish "Abstracts of Deaths at different ages in the different ranks and professions of society, in connection with an Abstract of the Ages of the persons following those professions, as returned at the last Census"—a work of great labour, involving the rearrangement of the Census-returns in correspondence with the Registration districts.

That the active exertions of the Registrar-General would receive the hearty co-operation of the class of the community best fitted to appreciate their importance, might be expected; and accordingly we are not so much surprised to find that the great mass of our profession has cheerfully aided in furnishing more accurate accounts of the Causes of Death, as we are disgusted at learning that some fifty persons have chosen to throw all the obstacles in the way their insignificant position furnished them with. We believe the Registrar-General possesses the power of compelling these crotchety personages to fulfil so obvious a duty to the public: but probably he is right in not enforcing obedience, on the ground that, "although they may have diplomas, it is probable that the information they would be induced to furnish would be of little comparative value, and might mingle errors among the facts spontaneously supplied by enlightened and accurate observers."

During 1843 and 1844 there were registered.

| | 1843 | 1844 |
|---|---|---|
| Marriages . . . . . | 123,818 | 132,249 |
| Births . . . . . | 527,325 | 540,763 |
| Deaths . . . . . . . | 346,446 | 356,950 |
| Excess of Births registered | 180,879 | 183,813 |

In the seven years 1838-44, 861,286 marriages, 3,556,649 births, and 2,437,922 deaths have been registered—the Census of 1841 showing a population of 15,912,773, which augmented by the excess of births over deaths would be increased, by the beginning of 1844, to 16,366,876, and by the beginning of 1845 to 16,550,689. This is, however, below the actual increase of population, which probably takes place at the rate of 1·335 per cent., giving 16,684,600, on January 1, 1845, and 17,000,000 before Midsummer 1846. " About 222,000 souls are added to the population of this part of the United Kingdom annually."

All the Marriages and Deaths since 1838 have been registered: but many Births have not. Why the Registration of Births was not rendered compulsory it would puzzle a conjuror to declare. Its not having been so much lessens the value of the returns for the early years; but the indefatigable exertions of the officers of late years, which the legislature might have well spared them, have now rendered the omissions comparatively insignificant. More *Marriages* were registered in 1844 than in any previous year. The number was 132,249. In 1843 it was 123,818, and but 118,825 in 1842. But so few had not occurred since 1832 as in 1842. From 1839 to 1842 they declined, increased again in 1843, and attained the maximum in 1844—corresponding strikingly with the degree of prevailing prosperity of the country. No less than 4·17 *per cent.* of the men and 13·16 *per cent.* of the women were under 21 years of age. 12·81 *per cent.* of the men, and 8·46 per cent. of the women married in 1844 had been previously married. The test of education contained in the power of *signing the marriage register*, has been so frequently alluded to of late, that our readers' may like to have the Registrar's opinion upon it.

" Only 67 in 100 men, and 51 in 100 women, wrote their names. It is probable that a few women, able to write letters intelligible to their friends, signed with marks; but this simple test leaves little doubt that 33 in 100 of the men and 49 in 100 of the women of England, at the marriageable age, are either quite unable to write, or write very badly. Some objections have been raised against this return as a test of the state of education. And it should be taken for no more than it is worth. I have already stated that a certain number of the women able to write, either from timidity or from other motives, may not have written their names. Upon the other hand, many who write their names are able to write little else; and writing the name is no proof of the possession of that stock of the elements of literary and scientific knowledge which it is desirable that the whole mass of a civilized nation should possess. But the return is of unquestionable value, as an evidence of the *relative state* of elementary education in different parts of the country, and at different times. It will be seen that there is a very slight diminution in the proportion of men who signed with marks during the six years 1839—44. The average age of men at marriage is about 27 years, and if the mean age of boys during their education be 10 years, the great bulk of the persons married in 1839—44 learned to write between the years 1821—7. The slow progress of instruction in those years is evinced by the facts, that 66·3 *per cent.* of the men wrote their names in the first, and only 67·6 *per cent.* (only 1·3 more) in 1844; while 50·5 *per cent.* of the women wrote their names in the first, and only 50·8 *per cent.* in the last year. I fear that the records of future years, in exhibiting the results of the inadequate means employed to educate the present generation of youth, will be as little flattering to our age as the actual returns are to our predecessors. The insufficiency of the national education is the more to be regretted, as the means of education exist, and the funds left for educational purposes, if properly applied, in the charities and public institutions, would, with some assistance from Parliament, supply the children of the poor with the sound knowledge which the scanty earnings of the parents do not enable them to purchase. The annual income of endowments for education is £ 312,544. The state of education varies in different counties to an incredible extent. [We extract a specimen of this from the tables. In the metropolis 12 *per cent.* signed with marks in 1844; in Cumberland, 16; in Cornwall, 36; in Lancashire, 40; in North Wales, 45; and in Bedfordshire, 50 *per cent.*]: and it will be observed, that in all counties, of any amount of population, the proportion of men and women who write remain very constant, from year to year, or vary slowly." P. 16.

" *Mortality.*—The annual mortality during 1838-44 was 2·189 per cent., or 1 in 46 of the population. It was above the average in 1838 and 1840; near it in 1839; lower in 1841 and 1842; and lowest in 1843, 1844. It varied from 1 in 43·7 (1840) to 1 in 47·2 1843. In the three first years (1838-40), the mortality was 2·239 *per cent.*; and in the three last (1842-4) 2·147, a fall of $\frac{1}{24}$ part. Out of an equal population, for every 24 deaths in the three first, there were only 23 in the three last years. The average price of wheat was 67s. 2d. the three first years, and 52s. 10d. in the three last. The average price of butcher's meat per cwt. paid at Greenwich Hospital was 48s. per cwt. in the three first, and 44s. 7d. in the three last years. The mean daily wages of bricklayers, masons, plumbers, and carpenters (of which an account has been kept at the hospital for some years), rose from 5s. 2d. a day, 1838-40, to 5s. 6d. in 1842-4. The declared annual value of British and Irish produce and manufactures exported from England and Wales was £47,138,173. in the three years 1838-40, and £48,400,977. in the three years 1842-4: the annual official value of imports in the corresponding years was £56,269,884. and £62,141,101. The annual amount of money expended in the relief of the poor in England was £ 4,581,600. in 1838-40, and £ 5,074,600. in 1842-4. In the last three years, therefore, the price of provisions was cheaper, the commerce and manufactures of the country more active, the relief to the destitute more liberally administered, and the wages of the artizan higher, than in 1838-40; and all these circumstances, favorable to the public health, undoubtedly contributed to the reduction of the mortality observed." P. 20.

Throughout the seven years, and in all the Registration-divisions of the country, the mortality of males has been greater than that of females. Throughout England, in 1844, to every 100,000 males living, there were 2236 deaths, while but 2074 female deaths occurred to every 100,000 females living.

Returns obtained from France, Prussia, and Austria, for 1843, give the following results :—

| To Persons living. | England. | France. | Prussia. | Austria. |
|---|---|---|---|---|
| One Marriage .. | 130 | 123 | 110 | 123 |
| One Birth ..... | 31 | 35 | 26 | 26 |
| One Death ..... | 46 | 42 | 37 | 33 |

M. Moreau de Jonnés has furnished detailed tabular statements of the mortality of *France* in 1843. From these it appears the total amount of deaths was 811,435 (the population being, according to the census of 1841, 34,230,178), viz. 406,432 male, and 405,003 female deaths. Of these deaths, 2606 took place from small-pox. The male suicides amounted to 1654, and the female to 488. There were 235 men and 71 women murdered; and 43 men, 2 women, executed. Accidental deaths amounted to 4,942 among the men, and 1494 among the women. The deaths from epidemic causes (the term being used in a different sense to that implied by it in the English returns) amounted to 1974, the proportion of the sexes being nearly equal.

The Government of *Austria* has likewise furnished some tabular statements, except as relates to Hungary, Transylvania, and the Military Frontier. Likewise excluding these portions of the territory, we find, from the census of Austria for 1840, that the population amounted to 20,975,258. The deaths in the year 1843 amounted to 697,342, viz. 355,518 male, and 341,824 female deaths. Of these 697,342 deaths, 669,889 are returned as *sporadic*, 10,054 as *endemic*, 6,847 as *epidemic*, 3,592 from *small-pox*, 890 from *suicide*, 40 from *hydrophobia*, 442 from *murder*, 5558 from *accidents*, and 30 from *executions*.

In reverting to the Returns for our own country, we regret we cannot furnish our readers with an account of the *Causes of Death* in 1843 and 1844, as abstracts of these are only contained in the present Report for these years, as far as regards the metropolis, the abstracts for England and Wales coming down only to 1842; and as they were published in the Report of last year (*See Med. Chir. Rev. July* 1845), we are at a loss to explain their reproduction here. The absence of these returns, and of Mr. Farr's usual letter commenting upon them, much impairs the utility of the present volume. The following is an extract from the Abstracts of the Causes of Deaths occurring in the Metropolis, its population being, in 1841, 1,915,104.

| *Metropolis.* | 1843. | | | 1844. | | |
|---|---|---|---|---|---|---|
| | Males | | Females. | Males. | | Females. |
| All Causes . . | 24961 | — | 23613 | 25729 | — | 24694 |
| Small-pox . . | 254 | — | 184 | 942 | — | 862 |
| Measles . . . | 759 | — | 683 | 627 | — | 555 |
| Scarlatina . . | 981 | — | 886 | 1545 | — | 1484 |
| Pertussis . . . | 863 | — | 1045 | 565 | — | 727 |
| Croup . . . | 219 | — | 168 | 218 | — | 193 |
| Typhus . . . | 1083 | — | 1000 | 880 | — | 816 |
| Hydrophobia . | 1 | — | 1 | 3 | — | — |
| Hydrocephalus . | 1097 | — | 711 | 982 | — | 781 |
| Convulsions . . | 1494 | — | 1207 | 1545 | — | 1191 |
| Bronchitis . . | 464 | — | 345 | 616 | — | 556 |
| Pneumonia . . | 2320 | — | 1904 | 2149 | — | 1915 |
| Phthisis . . . | 3733 | — | 3371 | 3750 | — | 3349 |
| Childbirth . . | — | — | 373 | — | — | 350 |
| Violent Deaths . | 763 | — | 355 | 865 | — | 436 |

We suspect the compilers of the present volume have been somewhat hard pushed for matter, inasmuch as they have re-inserted the "Statistical Nosology," which is already in the hands of every medical practitioner.

